# Exploring the Use of *Tenebrio molitor* Larvae Proteins to Functionalize Durum Wheat Pasta

**DOI:** 10.3390/foods14071194

**Published:** 2025-03-28

**Authors:** Serena Carpentieri, Agnieszka Orkusz, Joanna Harasym, Giovanna Ferrari

**Affiliations:** 1 Department of Industrial Engineering, University of Salerno, Via Giovanni Paolo II, 132, 84084 Fisciano, SA, Italy; scarpentieri@unisa.it; 2 Department of Biotechnology and Food Analysis, Wroclaw University of Economics and Business, 53-345 Wroclaw, Poland; agnieszka.orkusz@ue.wroc.pl (A.O.); joanna.harasym@ue.wroc.pl (J.H.); 3 Adaptive Food Systems Accelerator—Research Centre, Wroclaw University of Economics and Business, 53-345 Wroclaw, Poland; 4 ProdAl Scarl c/o, University of Salerno, Via Giovanni Paolo II, 132, 84084 Fisciano, SA, Italy

**Keywords:** novel protein sources, *Tenebrio molitor* larvae, durum wheat pasta, techno-functional properties, bioactivity

## Abstract

Background: Edible insects, such as *Tenebrio molitor* larvae (TM), offer a sustainable protein alternative to meet increasing dietary demands. The aim of this study is to investigate the functionalization of durum wheat pasta through the incorporation of TM flour (0–30%), focusing on how the addition of this non-conventional ingredient affects pasta production processing and its technological and chemical characteristics. Methods: Pasting properties, color, total phenolic content, antioxidant activity, and reducing sugars were determined for dry and cooked pasta. Texture profile and cooking properties were assessed for cooked samples. Results: The insect flour contributed to enhance polyphenols content in pasta, which increased from 0.06 and 0.03 mg_GAE_/g up to 0.19 and 0.10 mg_GAE_/g for dry and cooked pasta, respectively, and remained constant after the production process. The addition of TM flour altered the microstructure of wheat macromolecules, forming complex molecules, such as amylose–lipid complexes, and hydrogen and electrostatic interactions between proteins and polysaccharides, contributing to improved molecular stability and bioactivity. The pasta produced with insect flour up to 10% showed water absorption capacity, cooking properties, and consistency comparable to those of traditional pasta. Moreover, the addition of TM flour led to a reduction in peak viscosities from 2146.5 cP to 911.5 cP and roughness of pasta. Conclusions: The findings demonstrated the potential of TM flour as a unique source of bioactive compounds enhancing both the nutritional and functional properties of durum wheat pasta. Overcoming processing challenges through the optimization of product formulation and process parameters is crucial for exploring the production of insect flour enriched pasta at industrial scale while maintaining product uniformity and satisfying consumers expectations.

## 1. Introduction

Proteins are essential nutrients that play vital roles in metabolic processes, including growth, maintenance, and cellular repair. However, producing protein is resource-intensive and poses significant challenges to environmental sustainability. Therefore, to shift towards more sustainable and health-conscious diets, it is crucial to find alternative protein sources [1].

Nowadays, the consumption of edible insects has been proposed as a non-conventional solution to tackle issues related to the consumption of conventional animal proteins, such as food security, economic and environmental sustainability, and health-associated risks [2].

According to the Food and Agriculture Organization (FAO), food and feed production needs to increase by 60% by 2050 to face the needs of a growing population following a protein-rich diet [3]. To this purpose, insects are considered a low environmental impact protein source due to lower greenhouse gas and ammonia emissions and the reduced need of land and water compared to traditional proteins [1,4,5,6]; indeed, 15,000 L of water are needed to produce 1 kg of meat, ten times more than the water needed to produce 1 kg of cereal [7].

Insects possess higher nutritional value in comparison to cow meat, demonstrating their potential for introduction in human diet and in the food industry in response to the rising interest in nutrient-dense food options [3,8]. Insect-based proteins offer a comparable, and in some aspects superior, nutritional profile to conventional protein sources, alongside notable environmental benefits. The protein content of edible insects on a dry matter basis ranges from 35% for termites to 61% for crickets and grasshoppers, while conventional meats, such as beef, chicken, and pork typically contain 23–25% protein on a fresh weight basis [9]. Moreover, insects are rich in micronutrients such as iron, zinc, and B vitamins [10], as well as in unsaturated fatty acids, contributing to cardiovascular health [10].

Among insects, dried yellow mealworm larvae (*Tenebrio molitor*, TM) were the first species that obtained the authorization as a food ingredient in the European Union [8] and currently, it is one of the insect species most accepted by the consumers [11].

Studies revealed that larval forms of TM were on average 5% higher in protein content than meat chicken breast. This insect species contains omega 3, a sufficient content of vitamins and minerals, and present a favorable amino acid profile and a high ratio of polyunsaturated fatty acid (PUFA) and saturated fatty acid (SFA). Moreover, TM larvae possess good lipid nutritional indices, which contribute to its nutritional profile, making it a popular source of fat in various applications, including human and animal consumption [3]. When comparing TM to other insect proteins, several factors highlight its advantages in terms of nutritional value and functionality. Compared to other edible insects like crickets (*Acheta domesticus*), black soldier fly larvae, and locusts, TM offers a higher protein content (47–60% dry weight), with a more balanced amino acid profile, and a superior lipid composition, containing beneficial unsaturated fatty acids [12]. TM proteins exhibit better water and oil retention capacities than cricket and locust proteins, making them more suitable for pasta, baked goods, and meat alternatives [13]. TM proteins have also a high digestibility (~90%), similar to cricket proteins but superior to black soldier fly larvae, which contain a higher amount of chitin [12].

While there is a gradual increase in interest and product development in the insect-based food sector within Western markets, driven by nutritional benefits and environmental sustainability, several challenges such as potential allergenicity and consumer acceptance still need to be faced [14]. Indeed, the least liked protein-based food for the population are insects and cultured meat that follow single-cell-based and plant-based products, being the latter the most accepted [3]. Recent studies indicate that Western consumers’ acceptance of insect-based foods remains limited, primarily due to unfamiliarity and cultural barriers [15,16]. Western consumers are willing to consume insects when they are processed into “hidden” forms within food products. Therefore, to enhance acceptance, companies are incorporating insect proteins into familiar food items, such as burger patties, protein bars, and cookies [16]. Addressing cultural perceptions, enhancing product appeal, and implementing educational initiatives are essential steps towards broader consumer acceptance.

To encourage the consumers to integrate insects in their diet, especially in Italy where the culinary tradition is deeply rooted, a strategy to overcome the psychological barriers can be the combination of dried or grounded insects with staple foods like pasta [3,17]. The incorporation of TM flour into foods has been explored for bakery products such as bread [18,19,20,21], muffins [22], and biscuits [23,24], extruded snacks [25], and meat-based products [26,27].

To the best of our knowledge, only two studies were focused on investigating the incorporation of mealworm flour into egg pasta [28] and fresh wheat flour pasta [27]. Pasini et al. [29] investigated the fortification of “tagliatelle” pasta with insect protein fractions isolated from house cricket and yellow mealworm. Adding mealworm powder to pasta negatively affected both its cooking and sensory properties, including the color and texture [28,29].

The novelty of this study is the assessment of the effect of different formulations of TM flour and durum wheat semolina on the quality of dry pasta to select the optimal ratio of the two raw materials. In addition, it is essential to evaluate the effect of the drying and cooking processes on the quality of the dry pasta by simulating operating conditions similar to those utilized in the industrial pasta-making process to assess the industrial scalability of the process.

Therefore, this study aimed to investigate the effect of the addition of TM flour at different concentrations (5–30%, *w*/*w*) to durum wheat semolina on the quality of dry and cooked pasta. An extensive analysis was performed on both raw and cooked pasta to evaluate characteristics such as colorimetric and rheological properties, phenolic content, antioxidant activity, reducing sugar levels, texture, and cooking performance. This was aimed at identifying the best formulation for pasta production, with the goal of encouraging healthier eating habits while preserving the traditional consumption patterns of consumers.

Incorporating TM flour into durum wheat semolina could enhance pasta nutritional profile with minor sensory alterations (e.g., color and firmness). Consumer acceptance can then be improved by optimizing product formulation and processing conditions.

## 2. Materials and Methods

### 2.1. Raw Materials and Chemicals

Durum wheat semolina (*Triticum durum*) was purchased at Molino Spadoni (Ravenna, EM, Italy). The flour of yellow mealworm larvae (TM) was provided by Nimavert (Harelbeke, Belgium). A total of 100 g of TM powder contained 30.8 g of fats, of which 7.62 g was of saturated fatty acids, 6.7 g of carbohydrates, of which 2.0 g was of sugars, 50 g of proteins, 3.3 g of fiber, and 4 g of salt. Both the raw materials were stored in sealed bags at refrigerated conditions until use.

Solvents, all reagents, and standards used in the analyses were purchased from Pol-Aura, Zabrze, Poland.

### 2.2. Production Process of Durum Wheat Pasta

TM flour and durum wheat semolina mixtures (0%, 5%, 10%, 20%, 30% of insect flour and 100%, 95%, 90%, 80%, 70% of semolina), named as 0IN100S, 5IN95S, 10IN90S, 20IN80S, 30IN70S, were prepared in a rotary drum mixer (TM100, Vevor, Shanghai, China) operated for 10 min. The proximate composition, particle size, and the techno-functional characteristics of semolina, TM flour, and mixtures were previously reported by Carpentieri et al. [30].

Lab-scale pasta production equipment was used and standardization attempts were made for the potential scale-up of the process.

The mixing, kneading, and extrusion phases were performed in a pasta extruder (SIRMAN S.p.a., Pieve di Curtarolo, PD, Italy). The optimized conditions have been selected to reach a complete and uniform hydration of semolina. Water at 45 °C was added to each semolina/insect flour mixture to reach a moisture content of the dough of 32% in weight. The mixing speed was 60 rpm, and the mixing time was 15 min. The kneading speed was 100 rpm, the extrusion phase single-screw speed was 40 rpm, the extrusion pressure was 5–10 MPa, and the temperature of barrel zones was 40–55 °C to avoid protein denaturation.

A bronze die was used to produce pasta with “maccheroncini” shape. The increased surface roughness created on pasta by the bronze die improved its sensory characteristics, enhancing its ability to hold sauces due to increased surface area. Pasta extruded through bronze dies tends to have a more traditional, slightly uneven texture, which many consumers find more desirable as it gives the pasta a more authentic feel when cooked.

A tailor-made forced air convection chamber was designed and realized to carry out the pre-drying phase (trabatto). This phase (1 min, air temperature ≈ 80 °C) allows drying of the external surface of the pasta that loses 2–3% of moisture content, thus avoiding the sticking of the pieces and preserving the original shape. The drying step was performed using a static dryer (Biosec Pro, Tauro Essiccatori, Camisano Vicentino, VI, Italy). The drying process simulated the industrial production processing conditions for durum wheat pasta and it was designed by monitoring the moisture content of pasta and its physical characteristics d. The drying process consisted of 6 steps characterized by the specific temperature of the air (70–28 °C), relative humidity (70–50%), and drying time (90–40 min). The total drying time, including the cooling and stabilization steps, was equal to 7 h. The dry pasta, with a moisture content ≤12.5%, was then stored in sealed bags until use.

### 2.3. Flow Properties of the Blends

The flow properties of the investigated blends were determined using a ring shear tester Brookfield PFT (Brookfield Engineering Laboratories, Inc., Middleboro, MA, USA). The standard procedure used to operate the cell and reproduce the sequences of everyday stresses and the shear movement necessary to define the yield loci are described by Salehi et al. [31]. The tester operates by applying a vertical compression through the lid into the powder sample contained in the annular trough (internal volume 230 cm^3^, external annulus diameter 152.4 mm). A standard flow function test was conducted, during which the sample weight was recorded and input into the software. The axial speed of the lid approach movement and rotational speed of the cell were set to 1.0 mm/s and 1 rev/h, respectively. The test included 4 consolidation points and 5 over-consolidation points. The maximum everyday stress in all experiments was set to 4.846 kPa. The parameters measured were the unconfined failure strength (kPa), which is the stress required to cause a powder to flow at a stress-free surface after it has been compacted to a given consolidation level, the bulk density (kg/m^3^) as a function of the applied consolidation stress, the angle of internal friction which represents the friction between sliding layers of powder and defines the ratio of the major and minor principal consolidation stresses during steady state flow, and the cohesion (kPa), a measure of the strength retained by a powder after it has been compacted to a given consolidation level.

### 2.4. Pasting Properties

The rheological properties of dry and grinded pasta were determined by using a Rapid Visco Analyser (RVA 4500, Perten Instruments, Waltham, MA, USA), according to the official method AACC 76-21.01, as previously reported by Carpentieri et al. [30].

The peak viscosity (PV), trough viscosity (TV), breakdown viscosity (BV), final viscosity (FV), setback viscosity (SV), peak time, and pasting temperature were assessed.

### 2.5. Cooking Properties

The optimal cooking time (OCT) of pasta and cooking losses (CLs) was determined according to the official method AACC 66.50. Water absorption index (WAI), which is the weight increase in pasta after cooking, was determined and expressed as percent weight gain with respect to the uncooked pasta [32].

### 2.6. Texture Analysis

#### Cooked Pasta Adhesiveness and Hardness

The adhesiveness and hardness of the pasta cooked at the OCT was determined according to the official method AACC 66-52.01 using a texture analyzer (FC020STAV500, Axis, Gdańsk, Poland), equipped with a plexiglass cylindrical probe and a blade probe, respectively.

Compression cycles were carried out to attain 50% of sample deformation. Compression–decompression runs were performed at 100 mm/min to generate force–time curves. Adhesiveness (N) and hardness (N) values were calculated from the recorded data as the maximum positive force and maximum negative force, respectively. Six measurements were carried out per sample.

### 2.7. Colourimetric Parameters

A CR-310 colorimeter (Konica Minolta CR-310 chroma meter, Ramsey, NJ, USA) was used to determine the color parameters (lightness (L*), redness (a*), and yellowness (b*)) of dry and cooked pasta, according to the official method CIELab. The color difference (ΔΕ*) between each sample (made with semolina/insect flour mixtures) and durum wheat semolina pasta (control), and the color difference (ΔΕ*_dry-cooked_) between each dry and the corresponding cooked pasta samples was calculated according to Equation (1).

(1) $${\Delta E}^{*}=\sqrt{{\left(\Delta L^{*}\right)}^{2}+({\Delta a^{*})}^{2}+({\Delta b^{*})}^{2}}$$

### 2.8. Fourier Transform Infrared Spectroscopy (FTIR) Analysis

The Fourier Transform Infrared Spectroscopy (FTIR) spectra of dry and grinded pasta were recorded according to the methodology reported by Carpentieri et al. [30]. A FTIR spectrophotometer (Nicolet 6700 FT-IR, Thermo Fisher Scientific, Waltham, MA, USA) equipped with a diamond crystal cell for attenuated total reflection (ATR) operation was used.

### 2.9. Spectrophotometric Analyses

Dry and cooked pasta, previously lyophilized, were grinded by using a blender for 2 min. Briefly, 1.5 g of each sample were homogenized with 5 mL of a methanol/water (80:20 *v*/*v*) solution acidified with 1% HCl for 1 min in a vortex (MX-S, Chemland, Stargard, Poland). Then, the extraction was conducted by using a laboratory scale rotary shaker (MX-RD PRO, Chemland, Stargard, Poland) at room temperature for 2 h. The tubes were, then, centrifuged at 3500× *g* and 4 °C for 10 min (MPW-350, MPW MED. INSTRUMENTS, Warsaw, Poland). The collected supernatants were stored at −8 °C until use.

The same procedure, using water as extracting solvent, was followed for the determination of reducing sugars.

Total phenolic content (TPC), antioxidant activity via ferric reducing antioxidant power (FRAP assay), DPPH assay, and ABTS assay, and the reducing sugar content were determined using the methodologies previously described by Carpentieri et al. [30]. Results were expressed per gram of pasta in dry weight basis (g_DW_).

### 2.10. Statistical Analysis

All the experiments and analyses were performed at least in triplicate and the results were reported as means ± standard deviations. Differences among mean values were analyzed by one-way variance (ANOVA). The SPSS 20 (SPSS IBM, Chicago, IL, USA) statistical software was used. Tukey test, as a multiple comparison tests, was carried out to determine statistically significant differences between values for cooking and pasting properties, texture, color, and bioactivity (*p* < 0.05).

## 3. Results and Discussion

### 3.1. Flow Properties of the Blends

**Figure 1:**
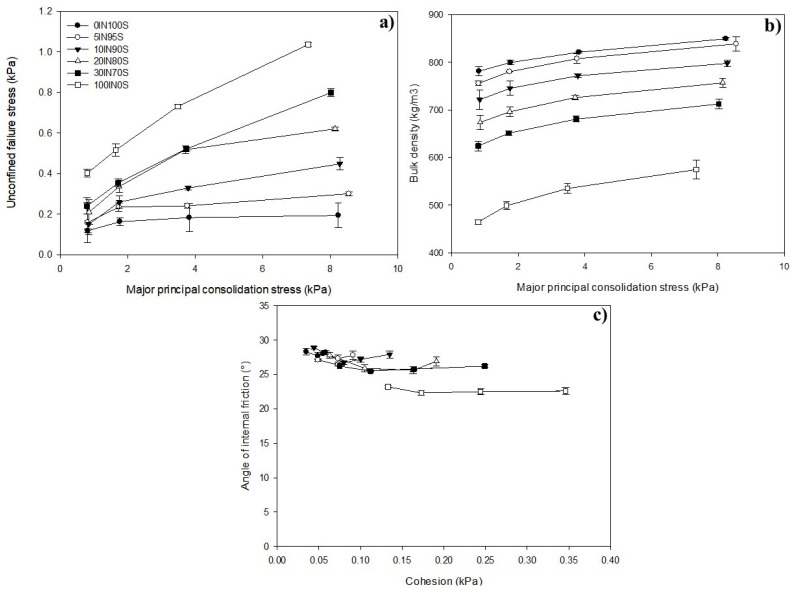
Flow properties of the mixtures analyzed: flow function (**a**), bulk density versus major principal consolidation stress (**b**), internal friction angle versus cohesion (**c**).

To understand how the addition of TM flour could modify the flowability of the semolina/TM mixture, the flow properties of the blends were measured. The uniaxial unconfined failure test is performed across different consolidation stresses and the flow function is constructed by plotting the unconfined failure stress versus the major principal consolidation stress, as shown in Figure 1a.

The flow function (ff), defined as the ratio between the consolidation stress σ1 and the unconfined yield strength σC [33], is frequently used to classify and compare powders’ flowability. A higher value of ff indicates better flowability, according to the following classification: ff < 1 non-flowing, 1 < ff < 2 very cohesive, 2 < ff < 4 cohesive, 4 < ff < 10 easy flowing, ff >10 free-flowing [33].

The semolina showed the highest ff (32.25), which gradually decreased with increasing the amount of insect flour in the mixtures (ff = 22.62 − 8.4 for binary mixtures) until reaching a value of 6.13 for insect flour. The ff values fell within the area of free-flowing powders, with only the sample 30IN70S and the insect flour showing an easy-flowing behavior.

The gradient of the lines used to fit the flow function curves is the flow index, typically in the range of 0.1–1. Lower values of the flow index suggest a better flowability [34]. The flow index values ranged from 0.031 for semolina to 0.16 for insect flour.

Figure 1b shows the dependence of the bulk density on the major principal consolidation stress for the samples tested. The bulk density increased with the consolidation stress due to the gradual accommodation of the particles [35].

The pressure exerted by the product on the bottom walls of a silo is significantly affected by changes in bulk density. As the bulk density of the stored material increases, the pressure applied to the silo structure also rises. Low fluctuations in the bulk density of approximately 8%, 9.9%, 9.6%, 11%, 12.4%, and 19.1% were observed for semolina, 5IN95S, 10IN90S, 20IN80S, 30IN70S, and insect flour, respectively. In line with previous findings [34], the most significant rise in bulk density was observed up to a specific stress level (4 kPa), suggesting that uncertainties in the project caused by variations in bulk density may be more significant in areas of the silo where lower consolidation stress occurs.

Figure 1c shows slight variations in the internal friction angle as a function of the cohesion of particles for the samples investigated. Results showed that consistently with the flow functions, with increasing the amount of insect flour in the mixtures, the cohesion increased. At the same time, the angle of internal friction gradually decreased starting from the internal friction angle of semolina (28.30°).

Changes in powder flowability, cohesion, and bulk density due to the addition of insect flour influence several key stages of industrial pasta manufacturing, from mixing and extrusion to drying and packaging. Durum wheat semolina is a coarse, free-flowing material, whereas TM flour is an easy flowing material due to its cohesiveness. Since this difference can lead to segregation issues during mixing, causing uneven distribution of insect flour in the dough, mixing times and speeds need to be optimized to achieve uniform flour dispersion, preventing inconsistency in protein content [36]. However, although the formulations investigated showed good flow properties, higher amounts of TM flour in the formulation might lead to clogging in automated feeders and hoppers, affecting ingredient dosing in industrial plants [37].

### 3.2. Pasting Properties

**Figure 2:**
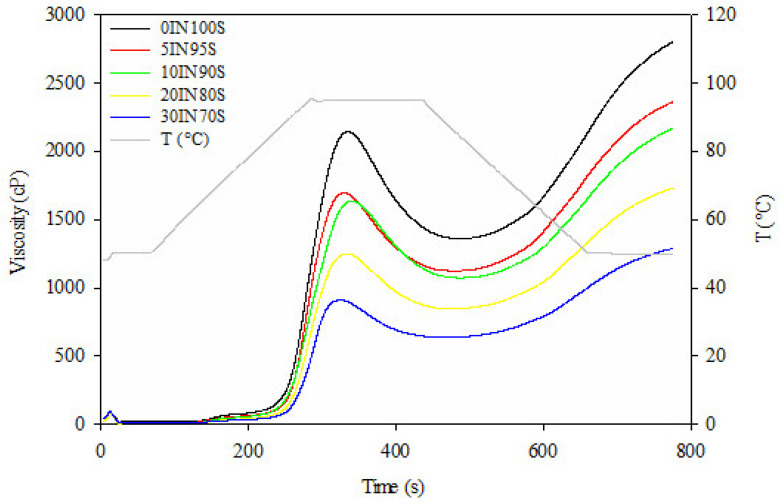
Pasting curves of dry pasta samples.

The pasting properties of a sample represent the modifications that occur inside it due to the application of heat in the presence of water and are influenced mostly by starch content in blends [38]. The obtained pasting curves are reported in Figure 2; the peak viscosity (PV), trough viscosity (TV), breakdown viscosity (BV), final viscosity (FV), and setback viscosity (SV) of the pasta samples are reported in Table 1.

The pasting curves (Figure 2) showed that all pasta samples enriched with insect flour had similar pasting profiles compared to the control pasta, although significantly lower pasting values were detected (Table 1).

The pasta samples’ PV, TV, BV, FV, and SV values underwent a significant decrease with increasing the amount of insect flour from 5% to 30%.

Peak viscosity indicates the ability of raw materials to absorb water and is related to starch swelling, being influenced mostly by starch content in blends. PV values of pasta enriched with insect flour decreased up to 57% when compared with the control pasta.

Consistently, Combrzyński et al. [38] demonstrated that increasing the amount of cricket flour into extruded snacks reduced peak viscosity due to the lower amount of wheat starch in the mixture. This suggested that the replacement of the starchy components with high-protein flour, rich in fats and fibers, that tend to compete with starch for water binding, results in lower water absorption and swelling capacity [38,39].

Moreover, it may indicate that a higher temperature is needed to transform blends with insect flour addition [38], which was confirmed by the pasting temperatures that showed a significant increase when the amount of insect flour was increased (Table 1). PT is the temperature at which the viscosity starts to increase during the heating process and indicates the starch resistance to swelling and rupture [40].

According to Nilsson et al. [41], the pasting temperature and viscosity decreased as protein replaced starch.

The breakdown viscosity gives a measure of the stability of the pasta during heating, reflecting the degree of starch granule disintegration and the resistance to withstand breakdown [38].

The insect-based pasta demonstrated a reduction in BV values up to 65% compared to the control pasta. Pasta samples enriched with 5% and 10% of insect flour showed no statistically significant changes in BV values.

An increased level of insect flour and protein content significantly lowered the susceptibility of starch granules to disintegrate during treatment, as also demonstrated by Villanueva et al. [42] and Combrzyński et al. [38].

The FV and SV values of the investigated samples showed similar trends. FV and SV values decreased when increasing TM flour percentage up to reach a reduction of 54% and 55%, respectively, when compared to the control pasta.

Likewise, SV values detected for the extruded snacks with 30% of cricked flour were two times lower than the control sample, showing a lower retrogradation tendency and rate that was attributable to a lower amount of starch in the sample [38].

TM flour is rich in hydrophilic proteins that compete with starch for binding to available water during gelatinization. This competition limits starch swelling, reducing pasting viscosity and overall gelatinization enthalpy. Insect-derived proteins may interact with starch via hydrogen bonding and electrostatic interactions leading to reduced swelling and lower peak viscosity, as the starch granules become less able to absorb and retain water. Proteins also reinforce the structure, reducing starch breakdown under heat and shear, which justifies lower final viscosity values in pasting profiles [24].

### 3.3. Cooking Properties and Texture Profile

The cooking properties in terms of OCT, CL, and WAI have been evaluated and the results obtained are reported in Table 2.

Results demonstrated that the cooking losses detected for pasta enriched with 5% and 10% insect flour were not significantly different from those for durum wheat pasta (control). High-quality durum wheat pasta should be characterized by cooking loss values not exceeding 7–8 g/100 g [43]. Therefore, it can be concluded that all the tested pasta formulations, except the pasta enriched with 20% and 30% insect flour, can be considered acceptable in terms of cooking losses. Interestingly, the CL values increased when adding 20% of insect flour and then, although still high, tended to decrease at 30% of insect flour. This tendency could be attributed to the increased amount of nonpolar compounds, which exceeded a threshold value and have hindered water absorption and amylose leakage. Nonpolar compounds, such as fatty acids, can interact with amylose to form complexes which prevent water absorption into the amylose cavity, thereby increasing the stability of the starch granules and reducing amylose leakage [44].

The amount of water absorbed per gram of dry pasta during the cooking phase of the different pasta samples has been determined and reported in Table 2. The main evidence found was that no significant differences were observed between the control pasta and the pasta samples enriched with 5% and 10% insect flour. However, in agreement with the values of pasting viscosities, WAI values significantly decreased when adding 20% and 30% of insect flour to the formulation.

Results also demonstrated that the weight of cooked pasta increased from 2.07 to 2.60 times with respect to the initial weight of dry pasta samples, in agreement with the expected ideal weight of cooked durum wheat pasta that should not exceed three times the dry weight [43].

These findings align with previous research on insect-enriched pasta and protein-fortified formulations, where optimizing the percentage of insect flour in the formulation is crucial to balancing nutritional benefits with acceptable cooking properties.

As it was outlined by Cabuk and Yilmaz [28], and consistently with the findings of the present study, insect flour enriched pasta (15%) needed more time to be cooked, exhibited lower volume expansion and water absorption, and higher OCT, but significantly improved protein contents in comparison to the pasta made with semolina. Other authors have shown that incorporating non-traditional flours into pasta formulations such as cricket flour generally led to a decrease in water absorption capacity [45]. This trend is likely due to the lower starch content and reduced gluten network formation, which limits the ability of pasta to retain water.

Likewise, the mealworm protein inclusion (14%) into pasta resulted in a darker pasta, with higher cooking losses [29]. Similar observations have been reported for pasta made with corn, rice, or legume flour, where higher cooking losses correlate with the lower gluten content [25].

**Figure 3:**
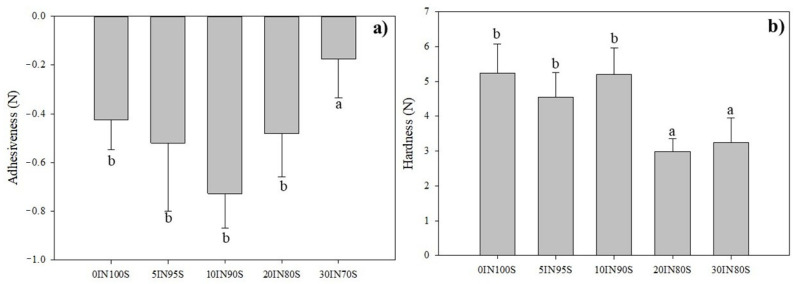
Adhesiveness (N) (**a**), and hardness (N) (**b**) of the cooked pasta samples. Values with different lowercase letters are significantly different (*p* ≤ 0.05).

Through the texture analysis it is possible to define the critical factors, such as process parameters, which affect the compactness and hardness of a food product and, consequently, its acceptability by consumers. In this study, the adhesiveness and the compression force needed to cut the pasta samples with the incisors were determined by a single compression analysis. The results obtained, depicted in Figure 3, demonstrated that the only statistically different value of adhesiveness was observed for the pasta enriched with 30% insect flour, which underwent a significant reduction compared to the other pasta samples. These changes can be related to the higher level of nonpolar lipid enrichment which induced the decrease in stickiness. In general, a greater adhesiveness is significant because of the higher presence of soluble substances on the surface of pasta after cooking. Higher lipid content and lower starchy compounds reduce the formation of amylose–lipid complexes leading to lower adhesiveness values [46]. Regarding the hardness, results showed that the addition of insect flour up to 10% did not exert any negative effect on the compactness of pasta after cooking, with cutting force values comparable to those of the control pasta. Further addition of insect flour to the formulation results in the absence of starch–lipid complexes, and few starch granules signally reduced the pasta hardness [46]. Generally, the addition of non-conventional ingredients to the durum wheat pasta increased cooking losses while reducing firmness due to the disruption of the gluten matrix and the lack of a well-developed gluten network resulting in softer pasta [47,48,49,50].

The addition of a higher amount of TM flour affects pasta texture by reducing firmness due to changes in gluten structure which may be perceived by the consumers as potentially unappealing.

Pasta enriched with TM flour also showed a darker color and a rougher surface, which can influence the sensory perception of the product quality. Moreover, TM flour has a nutty and slightly earthy flavor, which can subtly alter the taste of pasta. Depending on the proportion used, this change may be noticeable to consumers. However, at the lower insect flour content used (5–10%), the impact on taste and texture is minimal, while higher content (15% or more) may introduce stronger, more distinct flavors that could hinder consumers’ acceptance [24].

### 3.4. Color Parameters

The effect of replacing durum wheat semolina with increasing concentrations of TM flour (0–30%) on the colorimetric profile of dry and cooked pasta samples was investigated. The obtained results are reported in Table 3.

All the types of pasta showed high values of lightness (L*), in the range of 48–81. However, L* values significantly decreased in both dry and cooked pasta with increasing the amount of insect flour.

Dry and cooked pasta with 30% of insect flour showed the lowest L* values with a 38% and 32% reduction compared to the control pasta, respectively.

Instead, a significant increase in L* values (4–17%) was detected in the cooked pasta when compared to the corresponding dry pasta, especially for pasta with 20% and 30% of insect flour. This increase in lightness may be due to the cooking losses [51].

Regarding the redness values (a*) a gradual and significant increase (up to 3 times) was detected when increasing the amount of insect flour in both dry and cooked pasta compared to the control pasta. These visible changes are attributable to the presence of natural pigments which varies depending on diet, the growth stage of TM larvae, and environmental growing conditions. Melanin, which is the predominant pigment of mealworm larvae, is responsible for the brownish coloration of the exoskeleton. Moreover, mealworms accumulate carotenoids from their diet, including β-carotene, lutein, and zeaxanthin, leading to reddish or orange hues in their body [20,52].

Likewise, Çabuk and Yılmaz [28], who prepared traditional Turkish egg pasta by substituting wheat flour with mealworm flour, outlined that insect flour enriched pasta exhibited a darker color for the wheat flour pasta. Pasini et al. [29] stated that adding insect protein extract from *T. molitor* changed the color of the pasta which became similar to that of the whole semolina pasta.

Therefore, all the types of pasta enriched with insect powder showed color differences concerning the control pasta, as confirmed by the Δ*E* values (Table 3). Δ*Ε* values significantly increased for both dry pasta (14.16–29.98) and cooked pasta (10.32–26.01) with increasing the amount of insect flour from 5% to 30%. The highest ΔΕ value (29 on average) was detected for dry pasta functionalized with 20% and 30% of TM flour.

Δ*Ε* values evaluated between each dry pasta and the corresponding cooked pasta ranged from 3.06 and 10.67, indicating that the color difference increased as the amount of insect flour was increased and that the color changes are visible and detectable by human eyes [53]. However, consumers tend to perceive darker bakery and cereal-based products as healthier and higher in fiber or whole grains. Consequently, this change in color might enhance consumer interest in this type of product [54].

### 3.5. Fourier Transform Infrared Spectroscopy (FTIR) Results

**Figure 4:**
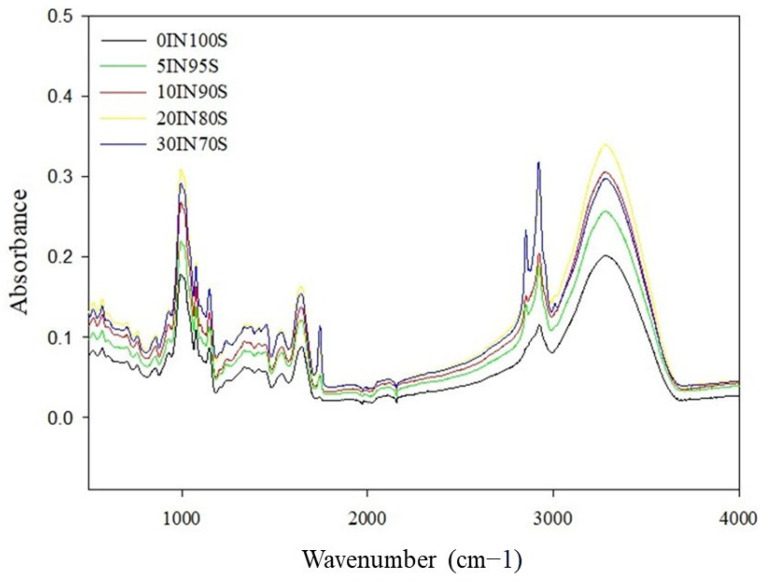
FTIR spectra of dry pasta samples.

The FTIR spectra of all dry pasta samples are reported in Figure 4. The samples exhibited a typical spectrum of durum wheat pasta as reported by Garcia-Valle et al. [55]. All the samples showed a similar spectrum with noticeable differences in their peak intensity, which increased as the amount of insect flour increased from 0% to 30%. In line with earlier studies, all spectra exhibited a prominent peak in the range of 3000 to 3500 cm^−1^ [30,55]. This peak is associated with the O-H stretching bond in carboxyl and hydroxyl groups and indicates the hydrogen bonds between water molecules and semolina and TM flour components. It is interesting to note the presence of a double peak like those reported by other authors [56,57] with higher absorbance values at 2850 and 2920 cm^−1^. This double peak, which was not detected in the traditional pasta sample, is associated with the aliphatic C–H stretching vibration of fatty acids and lipids, indicating the presence of methyl and methylene groups [58]. The intensity of this peak increased with increasing the amount of insect flour in the pasta samples, and this increase is particularly visible in the case of the pasta formulated with the 30% insect flour. The asymmetric and symmetric stretching associated with alkanes and alkenes exhibited higher absorbance at 2956 and 3257 cm^−1^ [56]. Pasta samples enriched with TM flour also showed a peak at 1750 cm^−1^, particularly evident in the pasta with the addition of 20% and 30% insect flour. This peak results from aliphatic unsaturated C=O vibrations of the esters of triacylglycerols [55,58]. All samples exhibited absorption peaks in the region of 1500 cm^−1^–1700 cm^−1^, indicative of amides I and II. The intensity of these peaks increased when the amount of insect flour increased [57]. In particular, the FTIR spectra revealed the presence of two broad absorption bands, identified as amide I and amide II, at 1630 cm⁻^1^, 1535 cm⁻^1^, and 1518 cm⁻^1^. These bands are typically used to analyze the secondary α-helix and β-sheet structures of proteins. They correspond to the C=O and C–N stretching vibrations of the peptide bonds [56]. The low intensity peaks detected at a wavenumber ranging from 1395 cm^−1^ to 1230 cm^−1^ correspond to the stretching vibrations of CH_2_ and CN bonds [57]. The “fingerprint” region of polysaccharides appears between 800 and 1200 cm^−1^. This range reflects the vibrations of CO, CN, CC, and CH groups. The C–O–C stretching modes, instead, appeared at 1150 cm^−1^ and 980 cm^−1^ [58].

Insect proteins like arginine- and lysine-rich proteins interact with wheat proteins through hydrogen bonds, electrostatic forces, and hydrophobic interactions [59]. This may alter the balance of β-sheet and α-helix structures in wheat gluten, affecting viscoelastic properties. Pasini et al. [29] found that incorporating insect flour to wheat pasta (up to 14%) led to changes in protein conformation, shifting the balance of β-sheets and α-helices, thereby modifying gluten functionality and suggesting structural modifications in the protein network due to new crosslinking interactions between insect and wheat proteins.

### 3.6. Bioactivity of Dry and Cooked Pasta Samples

**Figure 5:**
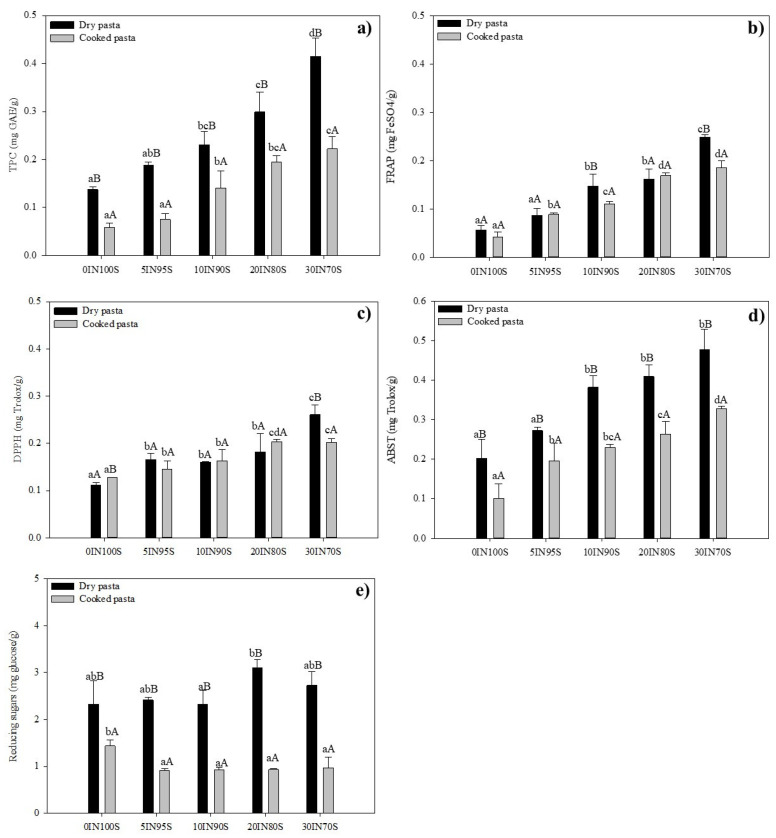
Total phenolic content (TPC) (**a**), ferric reducing antioxidant power (FRAP) (**b**), total antioxidant activity (DPPH) (**c**), total antioxidant capacity (ABTS) (**d**), and reducing sugar content (**e**) of the dry and cooked pasta samples. Values of pasta samples at increasing amounts of insect flour with different lowercase letters are significantly different (*p* ≤ 0.05). The values of the dry and cooked pasta samples at each amount of insect flour with different uppercase letters are significantly different (*p* ≤ 0.05).

The total phenolic content (TPC) and the antioxidant activity of the pasta samples are reported in Figure 5. Results demonstrated that replacing semolina with TM flour from 5% to 30% significantly increased the TPC and antioxidant activity of pasta. Durum wheat pasta usually possesses high functionality, with an average TPC of 0.14 mg GAE/g_DW_ of pasta, in agreement with the TPC previously detected for durum wheat semolina [30] and the TPC values found elsewhere for durum wheat pasta [39,60]. The dry pasta enriched with 30% insect flour showed the highest level of TPC (0.42 mg GAE/g_DW_). When the amount of insect powder was increased from 5% to 30%, TPC increased by 30% to 1.6 times compared to the control sample. A similar pattern was observed for the antioxidant activity throughout all the used assays. The pasta sample with the 30% of TM flour showed antioxidant activity values of 0.25 mg FeSO_4_/g_DW_, 0.26 mg Trolox/g_DW_, and 0.48 mg Trolox/g_DW_ for FRAP, DPPH, and ABTS assays, respectively. Pasta samples enriched with insect flour showed average values of antioxidant activity significantly higher than those detected for the traditional pasta (up to 2.5-fold for FRAP, 1.3-fold for DPPH, and 1.4 for ABTS). Anusha and Negi [56], who investigated the protein concentrate from TM to evaluate its suitability and applicability as a food ingredient, stated that the TPC showed exceptional antioxidant activity, with considerable DPPH and ABTS radical scavenging IC50 values. Tofu made with a 50% inclusion of either mealworm protein isolates or mealworm protein hydrolysate exhibited significantly enhanced antioxidant properties compared to tofu made from soybean flour [61]. Similarly, the addition of TM flour (3–6%) to biscuits [23], (10%) to muffins [22], and (10%) to ice cream [58] caused a significant increase in the antioxidant properties of these products. While there is limited research on the free-radical scavenging activity of products supplemented with insects, it has been reported that TM have strong antiradical properties due to its unique composition which includes redox ingredients able to counteract oxidative stress [30,62,63]. In addition to polyphenols, researchers have noted that the hydrophobic and aromatic amino acids found in mealworm proteins enhanced their antioxidant properties [58]. These proteins, along with polyphenol-linked proteins, usually formed through noncovalent and covalent interactions, also contributed to the scavenging activity of TM and increased the bioavailability of polyphenols [64]. To date, a limited number of studies addressed the addition of edible insects into pasta products. More research is needed, especially to investigate the stability of bioactive compounds in cooked pasta and how the steps of the pasta-making process affect the bioactivity of the final product. Kneading, extrusion, and drying processes can significantly reduce the total phenolic acids due to oxidizing reactions as well as polymerization reactions that make these compounds less bioavailable [65]. Interestingly, comparing the TPC and antioxidant activity of dry pasta samples (Figure 5) with the values detected in the mixtures used to produce these types of pasta [30], no drastic degradation phenomena were observed. During the cooking process, a significant loss of bioactive compounds can occur in pasta. Researchers indicated that cooking notably decreased the levels of phenolic compounds, isoprenoids, and carotenoids, likely due to their degradation at high temperatures or their release into the cooking water [66,67]. The obtained results, reported in Figure 5, showed that the level of TPC and antioxidant activity of pasta samples enriched with TM flour underwent significant degrading effects after the cooking phase. Specifically, the TPC level of the cooked pasta samples decreased about 45% and the antioxidant activity 15%, 10%, and 35% on average, for FRAP, DPPH, and ABTS, respectively, compared with the corresponding dry pasta samples. Interestingly, the decrements in TPC and antioxidant activity in cooked pasta detected in this study are lower than those found by other authors. Verardo et al. [68] studied the effects of the pasta-making process and cooking steps on total free phenolic compounds that decreased by about 74.5% in the cooked spaghetti. Likewise, Gull et al. [39] concluded that the TPC and antioxidant activity of cooked pasta samples decreased significantly by 51% and 55%, respectively, due to thermal degradation during cooking and the leaching of phenolic compounds into the cooking water. To understand the fate of these compounds, the hydrolysis of starch and the release of bioactive compounds from pasta during digestion need to be monitored. Studies have shown that the addition of non-traditional flours to pasta formulations like insect flours can reduce the digestibility of starch [69]. The reduction in gelatinization could result in lower starch digestibility, as not fully gelatinized starch is more resistant to enzyme breakdown in the digestive system. Likewise, higher fiber content has been associated with lower glycemic responses and can reduce the digestion rate of starch, leading to slower glucose absorption and a potentially beneficial effect on gut health [70]. Moreover, the digestibility of insect proteins in various food products has been found to be high, likely due to their amino acid profiles and lower anti-nutritional factors compared to certain plant-based proteins [71].

Dry traditional pasta and the dry pasta enriched with different amounts of TM flour and the corresponding cooked pasta samples showed similar values of reducing sugars. This can be due to the formation of a highly structured protein matrix developed during kneading and drying which enable to prevent the sugars leaching. In the case of cooked pasta, when adding the insect flour to the pasta formulations, the level of reducing sugars decreased significantly with respect to the control pasta (45% on average). It was demonstrated that the addition of edible insects to wheat-based products could lead to increased interactions between starch and protein by reducing the content of rapidly digested starch (RDS) associated with the generation of lower reducing sugars [23].

## 4. Conclusions

The replacement of durum wheat semolina with TM flour in the formulation of a staple foods such as pasta could be an opportunity for promoting the use of alternative protein sources without requiring consumers to drastically modify their typical diet.

This study was carried out to identify the potential use of TM flour in the formulation and production of unconventional durum wheat pasta to improve its nutritional value and antioxidant power. The mixtures investigated had good flow properties showing free-flowing and easy-flowing behaviors (32.25 ≤ ff ≤ 8.4).

The pasta samples enriched with TM flour (5–30%) showed higher redness (3-fold increase) and reduced whiteness with respect to the traditional pasta.

Similar pasting profiles were obtained for all the samples with reduced pasting viscosities, such as peak, trough, breakdown, final, and setback viscosities, up to 55% reduction on average when increasing the amount of insect flour, indicating improved starch granules stability.

The addition of insect flour contributed to the pasta bioactivity which was significantly enhanced in terms of total phenolic compounds and antioxidant activity and reduced the reducing sugars content in the cooked pasta. When the amount of insect powder was increased from 5% to 30%, TPC increased by 30% to 1.6 times compared to the traditional pasta.

This study showed that the pasta samples functionalized with the addition of TM flour up to 10% possessed water absorption capacity, cooking losses (<5.8 g/100 g), adhesiveness, and hardness values (5.2 N on average) comparable to those detected for the traditional pasta.

These findings indicated that pasta enriched with TM flour showed a higher amount of phenolic compounds while maintaining the physical characteristics of traditional pasta.

Notwithstanding the promising results obtained, further research is needed to understand the mechanisms of the interactions among the components of TM flour and semolina, and sensory evaluations of the product, including visual, olfactory, and tasting evaluations by the panelists, are of utmost importance to determine the acceptable level of insect powder addition.

Moreover, in vitro digestion studies, using a standardized static in vitro digestion method, are also needed to assess the potential beneficial effects of the newly formulated pasta as well as the digestibility of starch by evaluating the bioaccessibility of bioactive compounds and the starch hydrolysis during digestion.

A life cycle assessment (LCA) study should be conducted to evaluate the environmental impact of the entire production process of pasta enriched with TM flour, providing a comprehensive understanding of its sustainability.

Finally, the validation of the newly formulated pasta at the pilot scale should be carried out in view of the process scale-up and the industrial transferability of the results. Addressing flowability challenges, dough handling, and drying issues through technological innovation, optimized processes and formulations are essential for scaling up the production while ensuring product consistency and consumer acceptance.

## Figures and Tables

**Table 1:** Pasting parameters of dry pasta samples.

Sample	Peak Viscosity (cP)	Trough Viscosity (cP)	Breakdown Viscosity (cP)	Final Viscosity (cP)	Setback Viscosity (cP)	Peak Time (min)	Pasting Temperature (°C)
0IN100S	2147 ± 40 ^e^	1360 ± 28 ^e^	787 ± 12 ^d^	2802 ± 31 ^e^	1442 ± 3 ^e^	5.56 ± 0.05 ^b^	86.42 ± 0.04 ^a^
5IN95S	1699 ± 57 ^d^	1125 ± 36 ^d^	574 ± 21 ^c^	2362 ± 35 ^d^	1238 ± 1 ^d^	5.50 ± 0.04 ^b^	87.6 ± 0.5 ^bc^
10IN90S	1637 ± 13 ^c^	1074 ± 4 ^c^	563 ± 10 ^c^	2169 ± 5 ^c^	1095 ± 1 ^c^	5.67 ± 0.0001 ^c^	87.18 ± 0.04 ^ab^
20IN80S	1252 ± 22 ^b^	846 ± 13 ^b^	406 ± 9 ^b^	1733 ± 25 ^b^	887 ± 12 ^b^	5.53 ± 0.0002 ^b^	88.4 ± 0.6 ^c^
30IN70S	912 ± 11 ^a^	637 ± 6 ^a^	275 ± 4 ^a^	1289 ± 17 ^a^	653 ± 11 ^a^	5.37 ± 0.05 ^a^	89.65 ± 0.0001 ^d^

Values with different lowercase letters within the same column are significantly different (*p* ≤ 0.05).

**Table 2:** Optimal cooking time (OCT), cooking losses (CLs), and water absorption index (WAI) of pasta samples.

Sample	0IN100S	5IN95S	10IN90S	20IN80S	30IN70S
Cooking time = 1 min	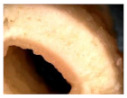	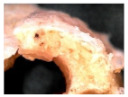	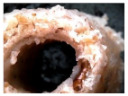	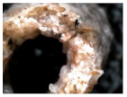	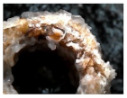
OCT (min)	10.0	11.5	12.0	15.0	16.5
	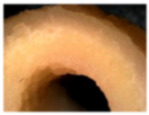	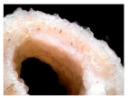	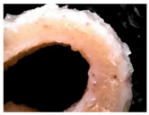	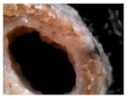	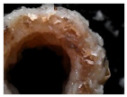
CL (g/100 g pasta)	5.7 ± 0.3 ^a^	5.0 ± 0.5 ^a^	5.6 ± 0.4 ^a^	9.5 ± 0.6 ^c^	7.2 ± 0.5 ^b^
WAI (g/g)	2.6 ± 0.4 ^b^	2.6 ± 0.2 ^b^	2.4 ± 0.2 ^ab^	2.2 ± 0.2 ^a^	2.1 ± 0.2 ^a^

Values with different lowercase letters within the same row are significantly different (*p* ≤ 0.05).

**Table 3:** Color coordinates (L*, a*, b*) of dry and cooked pasta as a function of the TM flour (0–30%). Color difference (ΔΕ*_dry_, ΔΕ*_cooked_) between each pasta and the durum wheat pasta (control) and color difference (ΔΕ*_dry-cooked_) between dry pasta and the respective cooked pasta.

Sample	0IN100S 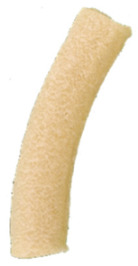	5IN95S 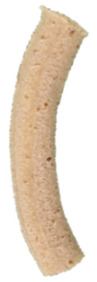	10IN90S 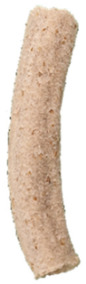	20IN80S 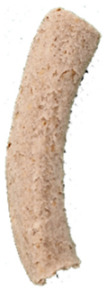	30IN70S 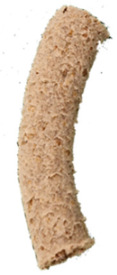
L*_dry_	77.79 ± 1.11 ^dA^	63.82 ± 2.85 ^cA^	56.044 ± 3.19 ^bA^	49.78 ± 2.60 ^aA^	48.067 ± 3.40 ^aA^
L*_cooked_	80.87± 1.86 ^eB^	70.82 ± 3.42 ^dB^	66.089 ± 1.94 ^cB^	59.96 ± 0.90 ^bB^	55.17 ± 3.15 ^aB^
a*_dry_	1.100 ± 0.10 ^aA^	3.37 ± 0.30 ^bB^	4.78 ± 0.62 ^cB^	4.74 ± 0.74 ^cA^	3.83 ± 0.60 ^bA^
a*_cooked_	1.089 ± 0.08 ^aA^	2.56 ± 0.15 ^bA^	3.38 ± 0.16 ^cA^	3.99 ± 0.45 ^dA^	4.44 ± 0.38 ^eA^
b*_dry_	15.18 ± 0.96 ^bA^	15.58 ± 0.91 ^bA^	17.31 ± 1.35 ^cA^	14.72 ± 0.95 ^bA^	12.40 ± 0.68 ^aA^
b*_cooked_	15.39 ± 0.84 ^aA^	17.27 ± 0.80 ^bB^	18.12 ± 0.56 ^bA^	17.83 ± 0.80 ^bB^	17.61 ± 1.40 ^bB^
ΔE*_dry_	/	14.16 ± 2.94 ^aA^	22.16 ± 2.73 ^bB^	28.25 ± 2.86 ^cB^	29.98 ± 3.10 ^cA^
ΔE*_cooked_	/	10.32 ± 2.64 ^aA^	15.20 ± 2.81 ^bA^	21.25 ± 2.50 ^cA^	26.01 ± 1.79 ^dA^
ΔE*_dry-cooked_	3.06 ± 2.00 ^a^	7.25 ± 2.50 ^ab^	10.17 ± 1.90 ^b^	10.67 ± 1.75 ^b^	8.83 ± 2.97 ^ab^

Values with different lowercase letters within the same row are significantly different (*p* ≤ 0.05). Values with different uppercase letters within the same column related to the same parameter are significantly different (*p* ≤ 0.05).

## Data Availability

The original contributions presented in the study are included in the article, further inquiries can be directed to the corresponding author.
